# Clinical Presentation, Management, and Outcomes of Primary Hyperparathyroidism during Pregnancy

**DOI:** 10.1155/2017/3947423

**Published:** 2017-09-25

**Authors:** Ya Hu, Ming Cui, Zhengyi Sun, Zhe Su, Xiang Gao, Quan Liao, Yupei Zhao

**Affiliations:** ^1^Department of General Surgery, Peking Union Medical College Hospital, Chinese Academy of Medical Sciences and Peking Union Medical College, Beijing 100730, China; ^2^Department of Obstetrics and Gynecology, Peking Union Medical College Hospital, Chinese Academy of Medical Sciences and Peking Union Medical College, Beijing 100730, China

## Abstract

**Background:**

Primary hyperparathyroidism (pHPT) in pregnancy is a rare event, but it poses a significant risk to mothers and fetuses. The optimal treatment strategy remains controversial.

**Methods:**

We present a consecutive series of twelve pregnant women with pHPT.

**Results:**

Twelve women were diagnosed with pHPT during pregnancy or in the postpartum period. Four of them presented no symptoms or mild symptoms. Four patients experienced serious complications, including hypercalcaemic crisis, acute pancreatitis, and eclampsia. Another four patients were identified postpartum as the result of neonatal convulsion with hypocalcaemia. Minimally invasive parathyroidectomy (MIP) under cervical plexus block was successfully performed in 11 of them during pregnancy or postpartum. The serum levels of ionized calcium and intact parathyroid hormone (iPTH) were much higher in patients with severe complications in this cohort than those in the group of patients with no symptoms or mild symptoms and patients who were diagnosed postpartum.

**Conclusions:**

MIP under cervical plexus block might be a safe and effective treatment for pregnant women with pHPT. Even though both conservative and surgical treatments are applicable for most mothers and fetuses with asymptomatic and mild hyperparathyroidism, serious complications may have catastrophic consequences for both.

## 1. Introduction

Primary hyperparathyroidism (pHPT) in pregnancy is a rare event, but it poses a significant risk to mothers and fetuses [1, 2]. For the mother, hyperparathyroidism can result in hyperemesis, nephrolithiasis, acute pancreatitis, or even hypercalcaemic crisis [3, 4]. For the fetus, maternal hypercalcaemia will increase the calcium levels in fetal serum and inhibit the fetal parathyroid axis, which may lead to intrauterine growth retardation, preterm delivery, intrauterine fetal demise, or postpartum neonatal tetany if the mother remains untreated [5, 6]. However, the diagnosis of pHPT during pregnancy can be difficult without the regular monitoring of serum calcium or parathyroid hormone. The main symptoms of pHPT during pregnancy are weakness, nausea, and vomiting, which are often misdiagnosed by patients or physicians as gestational physiological changes. This is also the possible reason for the wide variations in the reported prevalence in different studies. The application of medical treatment, such as calcitonin and bisphosphonates, is limited in pregnancy due to possible undesirable effects on the fetus, and surgery is considered the mainstay of management. The optimal timing of parathyroidectomy in pregnancy remains inconsistent in the literature [7]. Although surgery should be performed in the second trimester, some authors prefer to delay surgery until after birth due to concerns regarding the related impact on the fetus and the inability to use Tc-99m Sestamibi scans in pregnancy.

The literature contains some excellent reviews on this topic, and a consensus has been achieved to some extent [1, 8]. However, most of the reviews are case reports of patients with relatively mild disease; experience with patients who have serious complications, such as acute pancreatitis and preeclampsia, is limited. Thus, there is a need for a better understanding of the characteristics and natural history of pHPT during pregnancy. Here, we present a consecutive series of twelve pregnant women with hyperparathyroidism, which may be one of the largest published series from a single centre in recent years.

## 2. Subjects and Methods

An electronic database for pHPT in Peking Union Medical College Hospital, a tertiary referral university hospital, was retrospectively screened for pregnant patients from 2005 to 2016. The Ethics Committee of Peking Union Medical College Hospital approved this retrospective study, and written informed consents were obtained from the patients to use these data for research purposes. Clinical parameters, such as age at presentation, biochemical analysis, localization of lesions, pathological evaluation, surgical procedures, anesthesia, and fetal and maternal outcomes, were collected and summarized. All information on pregnancy complications, including abortions, preterm delivery, preeclampsia, eclampsia, and caesarean delivery, was also recorded (Table 1). A diagnosis of pHPT was made based on elevated serum levels of calcium and intact parathyroid hormone (iPTH), increased urinary calcium excretion, and histopathological confirmation of removed parathyroid lesions.

The nonparametric Mann–Whitney *U* test was used for intergroup comparisons of continuous variables. *p* < 0.05 was considered statistically significant. SPSS version 16.0 for Windows (SPSS, Chicago, IL, USA) was used for all analyses.

## 3. Results

In the past 11 years, a total of 1061 female patients were diagnosed with pHPT and underwent parathyroidectomy under cervical plexus block in this institute. Twelve patients were diagnosed with pHPT during pregnancy or in the postpartum period. The demographics, symptoms, clinical biochemistry, complications, and outcomes of these patients are summarized in Table 1. The average age of the pregnant patients was 29.8 years (range, 22 to 39 years). The serum iPTH level ranged from 137 to 1776 pg/mL (normal range: 12–65 pg/mL). The serum calcium level and urinary calcium excretion at 24 hours after presentation exceeded the upper normal limit in all 12 patients.

The clinical presentation in the present cohort varied from an absence of symptoms and nonspecific mild symptoms to hypercalcaemic crisis and serious complications. According to the clinical presentation and treatment history, the patients in the present cohort were divided into the following three diagnostic groups: mild hyperparathyroidism in 4 patients, severe hyperparathyroidism with life-threatening condition in 4 patients, and diagnosis from neonatal tetany in the postpartum period in 4 patients.

Eight patients were diagnosed before delivery. Four patients (cases 1, 2, 3, and 4) had relatively mild hyperparathyroidism, two of whom complained of nausea and vomiting (cases 3 and 4), and the other two were asymptomatic (cases 1 and 2). After receiving primary medical treatment for hydration and maintaining a low-calcium diet, their serum calcium levels decreased and their symptoms were effectively relieved. Three patients (cases 2, 3, and 4) underwent MIP during pregnancy from 16 to 40 weeks gestation after the lesion was localized by cervical ultrasound scanning alone. One patient (case 1) with multiple endocrine neoplasia type 1 (MEN-1) refused to undergo parathyroidectomy due to the potential adverse effects on the fetus. For all 4 patients, uneventful deliveries occurred at term, and the neonates were healthy with normal serum calcium levels.

Four pregnant women (cases 5, 6, 7, and 8) suffered from severe hyperparathyroidism with serious complications, including hypercalcaemic crisis in 3 patients (cases 5, 7, and 8), acute severe pancreatitis in 1 patient (case 5), eclampsia in 1 patient (case 6), and loss of consciousness in 3 patients (cases 5, 6, and 8). Emergent artificial abortion was required in three mothers (cases 5, 6, and 8) to relieve eclampsia or acute severe pancreatitis after systematic conservative treatments failed. Serum calcium levels decreased after parathyroidectomy. Another patient (case 7) with hypercalcaemic crisis was referred to our institute with a total serum calcium level of 4.5 mmol/L and an ironized calcium level of 3.07 mmol/L. The medical treatment, including hydration, diuretics, and calcitonin, could not decrease the calcium level, and bisphosphonates were ultimately used. MIP was performed successfully after a solitary parathyroid lesion was identified by ultrasound examination. Afterward, induced abortion was applied in midterm pregnancy according to the patient's wishes.

Four patients (cases 9, 10, 11, and 12) were not diagnosed until the postpartum period in the present series, and as a result, their babies suffered from convulsions due to hypocalcaemia. After the involved parathyroid was localized by Sestamibi scanning and cervical ultrasound examination, MIP was successfully performed.

**Figure 1:**
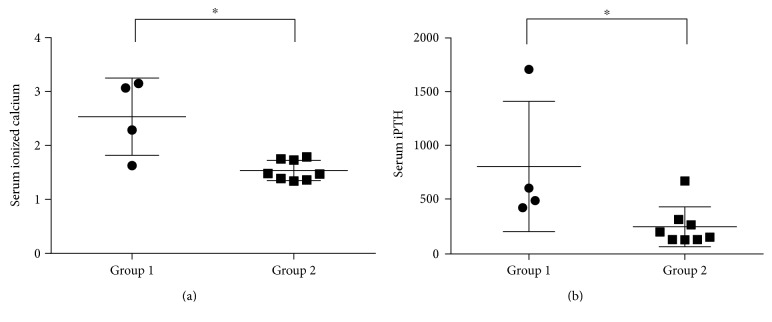
Comparisons of the preoperative serum level of ionized calcium and iPTH between patients with pHPT during pregnancy with severe complications (group 1) and those with mild hyperparathyroidism or who were diagnosed postpartum (group 2). (a) The serum level of ionized calcium was significantly higher in group 1 than in group 2 (Mann–Whitney *U* test, *p* = 0.0283, ^∗^*p* < 0.05). (b) The serum level of iPTH was significantly higher in group 1 than in group 2 (Mann–Whitney *U* test, *p* = 0.0263, ^∗^*p* < 0.05).

Compared with the group of patients who were diagnosed postpartum and the patients with mild hyperparathyroidism, the serum levels of ionized calcium and iPTH were much higher in patients with severe complications in this cohort (Figure 1). No significant difference was found between them in tumour size or maternal age at presentation (Table 2).

MIP was performed in all patients, except the one with MEN-1. Under ultrasound guidance, combined deep and superficial cervical plexus blocks were performed with ropivacaine. A satisfactory analgesic effect was achieved for all these patients under cervical plexus block and intravenous sedation during surgery. With a 2 to 4-centimetre incision, all surgical procedures were completed under direct visualization with a mean operative time of 27.3 (21–32) minutes. Solitary parathyroid lesions were present in 11 patients. Parathyroid carcinoma was identified in one patient after pathological evaluation following MIP, who was under close follow-up. Neck ultrasound examination successfully identified the parathyroid lesions before surgery. All hyperfunctional parathyroid lesions were removed successfully without related complications, except transient hypocalcaemia after surgery, which were relieved by oral supplementation with calcium and vitamin D. The patients had an uneventful recovery and were discharged on the second postoperative day. No recurrence of hyperparathyroidism was observed after regular monitoring of serum iPTH and calcium levels during a median follow-up period of 44 months (range: 8–131 months). No evidence of abnormal growth and development was present in these babies.

## 4. Discussion

It was reported that the incidence of pHPT is lower than 1% in pregnancy, while most cases of pHPT occur in elderly women [2, 7]. pHPT in pregnancy was first described in 1931, but there is no universal consensus regarding management due to its low incidence. Most reports in the literature are about single cases or small case series, and further experience in diagnosis and management is still necessary. The present report of twelve cases of pregnant women with pHPT illustrates the different characteristics of this rare condition.

The literature shows that up to 80% of pregnant women with pHPT are asymptomatic [9]. The serum calcium level was decreased by the physiological changes of pregnancy [10]. Although serious consequences, including hypercalcaemic crisis, acute pancreatitis, and eclampsia, may be life-threatening, only limited cases have been reported in the literature. The incidence of hyperparathyroidism in China is lower than that in Western countries; thus, the underdiagnosis of pHPT is likely, especially in remote rural areas of this country. Parathyroid hormone and ionized calcium are not affected in pregnancy, but pregnant women do not undergo routine testing of these levels in many areas [11]. Therefore, pregnant women with serious complications of pHPT are frequently referred to our institution.

Four patients in this cohort suffered from hyperparathyroid crisis, eclampsia, or acute severe pancreatitis. Preeclampsia is a disorder of pregnancy characterized by hypertension and proteinuria. In severe situations, it may result in eclamptic seizures, which are likely to require delivery of the fetus and placenta by the induction of labour or caesarean section. Hyperparathyroidism is a risk factor for preeclampsia [12]. It was reported that the risk of preeclampsia in pregnant women with parathyroid adenoma is approximately 6-fold higher than that in pregnant women without this disease [13]. The underlying mechanism is not fully understood, but a disorder of calcium homeostasis was believed to be an important aetiology for preeclampsia. Since 1995, 6 cases of preeclampsia in patients with pHPT have been reported in English publications and no eclampsia developed. Due to immediate surgical intervention or close follow-up, all babies survived, even though premature delivery was common. In contrast, one patient in the present cohort presented with eclampsia, and an immediate termination of pregnancy was mandatory after medical therapy failed. Acute pancreatitis in pregnancy is a rare and difficult condition in patients with pHPT in pregnancy and has been reported in 8 case reports since 1995 in addition to our report [14]. Even though the incidence is low, acute pancreatitis should always be included in the differential diagnosis for patients with abdominal pain during pregnancy.

In our series, 4 patients were diagnosed in the postpartum period because of neonatal tetany. It was reported that neonatal hypocalcaemic tetany may be present in approximately 50% babies of untreated mothers [15]. In pregnancy, the maternal calcium shunts through the placenta to the fetus, while PTH cannot transfer through the placenta. The elevated calcium levels in the fetus suppress the fetal parathyroid glands and relieve the maternal hypercalcaemia. After delivery, the calcium infusion to the fetus is stopped abruptly, and the neonatal calcium level is not maintained by the suppressed parathyroid glands. The neonate may suffer from hypocalcaemic tetany, while hypercalcaemia may worsen dramatically in the mother. Accordingly, for pregnant women treated medically to term, hypercalcaemic crisis in the mother and hypocalcaemic tetany in the neonate should be monitored carefully during the postpartum period.

There is no universal consensus regarding the management of pHPT during pregnancy due to the rarity of the situation. Medical treatment and close monitoring of the mother and fetus were suggested by some experts in cases of mild hypercalcaemia, while other authors believed that their outcome after surgery was more favourable [16]. Two studies based on community data by Hirsch et al. and Abood and Vestergaard showed that the abortion rate was not related to either maternal pHPT or delayed surgery, while a study conducted in a referral centre by Norman et al. demonstrated that the abortion rate was 48% among 32 pregnant women who did not undergo parathyroidectomy [5, 7, 16]. The possible reason for this discrepancy was speculated to be the selection bias in these studies. The management should be personalized to achieve a balance between the risk of hypercalcaemia and the risk of surgery and anesthesia [16, 17]. Surgical intervention was suggested by some authors for women with pHPT with a serum calcium level above 2.75 mmol/L during pregnancy [6, 18]. We also found that the severe complications of pHPT were related to a high serum level of calcium and iPTH. Therefore, surgery should be considered if primary medical management cannot control blood calcium satisfactorily.

The debate regarding the use of general or regional anesthesia during pregnancy is well known. Historically, general anesthesia and nonobstetric surgery during pregnancy were reported to be related to a higher incidence of prematurity and intrauterine growth retardation [19] and abortion [20, 21]. However, recent clinical data suggested that general anesthesia in pregnant patients may not have as much potential for harm as previously thought [22, 23]. Regional anesthesia is another feasible option for pregnant patients [24, 25]. Although almost all patients with MIP in pregnancy were reported to receive general anesthesia, based on a tradition that has been proven effective, we prefer to perform a MIP under a cervical plexus block in pregnant women in our institution [26]. To the best of our knowledge, this report documents the first series of pregnant women with pHPT who underwent MIP with cervical plexus block. The cervical plexus block can provide a satisfactory anesthetic effect for surgery, while it should be performed by experts in a closely coordinated multidisciplinary team [27]. Furthermore, the potential for toxicity related to local anesthesia in the doses used is unclear and requires further study. MIP has become the main treatment for pHPT with a single lesion over the past 2 decades and can also be performed in pregnant women [28–30]. More than 85% of pregnant women with pHPT suffer from one solitary parathyroid adenoma [14, 17]. MIP is preferred over traditional four-gland exploration due to the advantages of less trauma, a shorter operative time, and less anesthesia interference, which is important for the fetus. Preoperative imaging evaluation to localize the affected parathyroid gland is essential. Due to radioactive exposure to the fetus, 99mTC-Sestamibi scanning and computed tomography are restricted, and ultrasound is the first-line option for patients with pHPT in pregnancy. The sensitivity and specificity of ultrasound examination for the identification of parathyroid lesions were reported to be 68% and 94%, respectively, while the performance was highly dependent on the experience of the operators [31, 32]. For ultrasound negative patients, 4D CT scan may be relatively safer than sestamibi scan as lead shielding of the abdomen could mitigate potential radiation exposure to the fetus. As above, a multidisciplinary team with close cooperation between the surgeon, anesthesiologist, endocrinologist, radiologist, and obstetrician is critical in the perioperative care of this unique group of patients.

A major limitation of this study is its retrospective design, with inevitable selection bias. Besides, the sample size is still too small to reveal a statistically significant effect of the intervention or rare complications. Because of the rarity and potentially ethical considerations of this situation, it seems difficult to perform prospective controlled studies. Multicentre studies might be feasible to draw more convincing conclusions and improve diagnosis and treatment for pHPT during pregnancy.

In conclusion, pHPT during pregnancy is a rare condition associated with a significant risk of maternal and fetal complications. Conservative treatment or MIP under cervical plexus block might be safe for most mothers and fetuses with no or mild symptoms. However, serious complications, such as hypercalcaemic crisis, acute pancreatitis, and eclampsia, may have catastrophic consequences for both the mother and fetus.

## Figures and Tables

**Table 1:** Summary of clinical information of patients with pHPT during pregnancy.

Number	Age at presentation (years)	Gestation (weeks)	Symptoms	Obstetric history	Complications	Hypercalcaemic crisis	Eclampsia	Tumour size (diameter, cm)	Maternal serum iPTH (pg/mL)	Maternal serum ionized calcium (mmol/L)	Fetal outcome	Pathological result
(1)	37	7	None	G5P1A3	No	No	No	/	137	1.34	Normal	/
(2)	31	25	None	G1P0	No	No	No	2.9	213	1.79	Normal	Atypical adenoma
(3)	28	11	Nausea and vomiting	G1P0	No	No	No	2	162	1.75	Normal	Atypical adenoma
(4)	29	5	Nausea and vomiting	G1P0	No	No	No	1.5	139	1.73	Normal twins	Adenoma
(5)	32	27	Abdominal pain, diarrhoea, and loss of consciousness	G1P0	Acute pancreatitis, nephrolithiasis, and gestational hypertension	Yes	No	1.8	514	2.29	Artificial abortion	Adenoma
(6)	22	24	Headache and loss of consciousness	G1P0	Gestational hypertension	No	Yes	2.6	446	1.63	Artificial abortion	Adenoma
(7)	35	18	Nausea and vomiting	G2P0A1	Nephrolithiasis	Yes	No	3.1	634	3.07	Artificial abortion	Adenoma
(8)	34	6	Vomitingand loss of consciousness	G6P1A4	Nephrolithiasis	Yes	No	4.8	1776	3.15	Artificial abortion	Adenoma
(9)	26	Postpartum	None	G1P1	No	No	No	1.9	280	1.39	Hypocalcaemic tetany	Hyperplasia
(10)	28	Postpartum	No	G1P1	No	No	No	1.6	702	1.47	Hypocalcaemic tetany	Adenoma
(11)	26	Postpartum	Backache and vomiting	G1P1	Nephrolithiasis and gestational hypertension	No	No	2.7	333	1.48	Hypocalcaemic tetany	Adenoma
(12)	29	Postpartum	No	G1P1	No	No	No	3.1	139	1.36	Hypocalcaemic tetany	Adenocarcinoma

**Table 2:** Comparisons of clinical information between patients with pHPT during pregnancy with severe complications (group 1) and patients with mild hyperparathyroidism or those with a postpartum diagnosis (group 2).

	Group 1	Group 2	*p* value
Maternal age (years)	30.8 ± 6.0	29.3 ± 3.5	0.4343
Tumour size (cm)	3.2 ± 1.2	2.1 ± 0.7	0.1051
Serum ionized calcium (mmol/L)	2.54 ± 0.72	1.54 ± 0.19	**0.0283**
Serum iPTH (pg/mL)	842.5 ± 627.2	263.1 ± 191.7	**0.0263**

All results are expressed as the mean ± SD.
